# Photoreceptor Impairment and Restoration on Optical Coherence Tomographic Image

**DOI:** 10.1155/2013/518170

**Published:** 2013-04-03

**Authors:** Yoshinori Mitamura, Sayaka Mitamura-Aizawa, Takashi Katome, Takeshi Naito, Akira Hagiwara, Ken Kumagai, Shuichi Yamamoto

**Affiliations:** ^1^Department of Ophthalmology, Institute of Health Biosciences, The University of Tokushima Graduate School, Tokushima 770-8503, Japan; ^2^Department of Ophthalmology and Visual Science, Chiba University Graduate School of Medicine, Chiba, Japan

## Abstract

With recent development of spectral-domain optical coherence tomography (SD-OCT), the pathological changes of retina can be observed in much greater detail. SD-OCT clearly delineates three highly reflective lines in the outer retina, which are external limiting membrane (ELM), photoreceptor inner and outer segment (IS/OS) junction, and cone outer segment tips (COST) in order from inside. These lines can serve as hallmarks for the evaluation of photoreceptor condition. In retinitis pigmentosa (RP) leading to photoreceptor degeneration, the ELM, IS/OS, and COST lines are shortened with the progression of the disease. In addition, shortening of the ELM, IS/OS and COST lines is significantly associated with each other. The line length is longest in the ELM, followed by the IS/OS, and COST, suggesting that retinal layer becomes disorganized first at the COST, followed by the IS/OS and finally the ELM. This finding is consistent with the previous report that the earliest histopathological change in RP is a shortening of the photoreceptor outer segments. On the other hand, retinal layer becomes restored first at the ELM, followed by the IS/OS and finally the COST after macular hole surgery. There may be a directionality of photoreceptor impairment or restoration on optical coherence tomographic image.

## 1. Advancement of Optical Coherence Tomography Instrument

Optical coherence tomography (OCT) is a well-established method of examining the retinal architecture *in vivo*. After the introduction of OCT, *in vivo* imaging of the retina of various retinal diseases came true. In particular, the ease with which these images can be acquired considerably changed the diagnostic strategy used by ophthalmologists [1].

With most recent development of spectral-domain OCT (SD-OCT), the pathological changes of retina can be observed in much greater detail. SD-OCT technology uses low-coherence interferometry to detect light echoes, relying on a spectrometer and high-speed camera and based on the mathematical premise of the Fourier transformation [2]. Because application of the Fourier transformation has the effect of measuring all echoes of light simultaneously, as compared with sequentially in the case of time-domain OCT (TD-OCT), SD-OCT significantly increases the amount of data acquired in each session, resulting in a significant reduction of motion artifacts and an increased signal-to-noise ratio compared with TD-OCT. The axial resolution of TD-OCT was 10–20 *μ*m. SD-OCT (5 to 6 *μ*m of axial resolution) has improved the ability to detect intraretinal microstructures and to identify pathological changes in the retinal architecture in various diseases.

SD-OCT clearly delineates three highly reflective lines in the outer retina, which are external limiting membrane (ELM), photoreceptor inner and outer segment (IS/OS) junctions, and cone outer segment tips (COST) in order from inside. These lines can serve as hallmarks for the evaluation of photoreceptor condition [3]. In this review, we summarize the changes of these three lines on the SD-OCT images due to the photoreceptor impairment and restoration.

## 2. Photoreceptor Impairment on Optical Coherence Tomographic Image

Retinitis pigmentosa (RP) is a slowly progressive inherited retinal disease, and the patients experience reduced visual function because of the degeneration of the photoreceptors and the retinal pigment epithelium (RPE). The loss of the central photoreceptors reduces central vision at the end stage of the disease. In OCT, retinal edema or hemorrhage may weaken the signal intensity of the outer retinal layers, making it difficult to detect the reflections from the ELM, IS/OS, or COST [4]. In Japanese RP patients, however, it has been reported that macular abnormalities such as cystoids macular edema or epiretinal membrane were detected by OCT in only 7.4% of 622 eyes with RP [5]. Therefore, RP is a good candidate for image assessment of the photoreceptor impairment on the OCT images.

In RP patients, the IS/OS line disappeared from peripheral part toward fovea on the OCT images with the progression of the disease. Therefore, measuring the length of the residual IS/OS line can be useful in estimating the residual central visual function in RP patients. Using TD-OCT, it has been reported that the length or the presence of the IS/OS line was significantly correlated with visual acuity in 300 eyes with RP [6]. In addition, the length of the IS/OS line was highly correlated with the retinal sensitivity in RP patients [7]. It has also been reported that the length of the IS/OS line decreased with the progression of RP during the follow-up period of more than 2 years [8]. Shortening of the IS/OS line was accompanied by a decrease in retinal sensitivity and a worsening of the visual acuity. The decrease in IS/OS length was significantly correlated with the decrease in retinal sensitivity and visual acuity [8]. These results indicate that a progressive IS/OS shortening may reflect morphological changes of the photoreceptors and worsening of visual function in the progression of RP. Thus, the IS/OS line may be an important parameter to monitor in RP patients.

**Figure 1:**
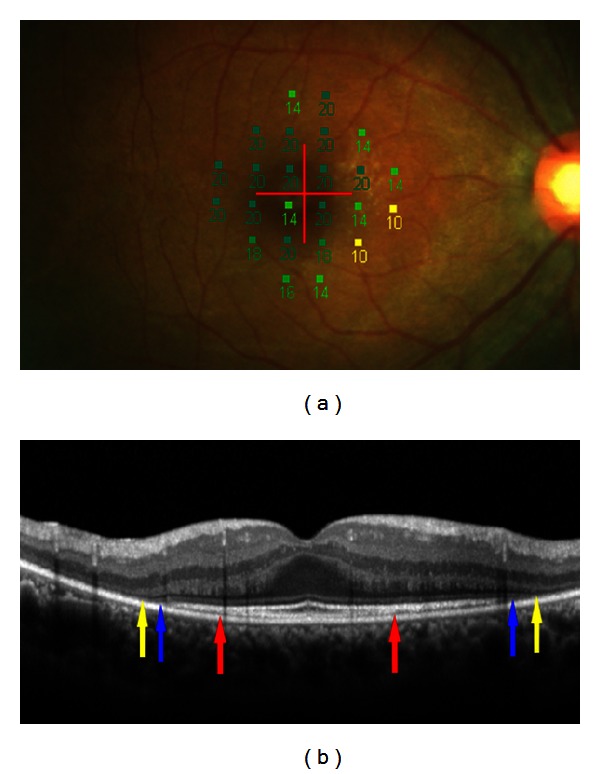
Microperimetric map and spectral-domain optical coherence tomographic (SD-OCT) image of an eye with retinitis pigmentosa (42-year-old woman). (a) Microperimetric map image. A total of 24 stimulus locations covering the central 10° field were tested. The mean retinal sensitivity at the 24 locations is 17.3 dB. The best corrected visual acuity was 1.0. (b) SD-OCT image of a vertical scan. In addition to photoreceptor inner/outer segment (IS/OS) junction line, the external limiting membrane (ELM) and cone outer segment tips (COST) lines are clearly seen on the SD-OCT image. Red arrows indicate end points of the COST line. Blue arrows indicate end points of the IS/OS line and yellow arrows end points of the ELM line. The lengths of the COST, IS/OS, and ELM lines were computed at 2272, 4629, and 4930 *μ*m, respectively. Note that the line length was the longest in the ELM, followed by the IS/OS and COST. The direction of photoreceptor impairment is from outside toward inside.

**Figure 2:**
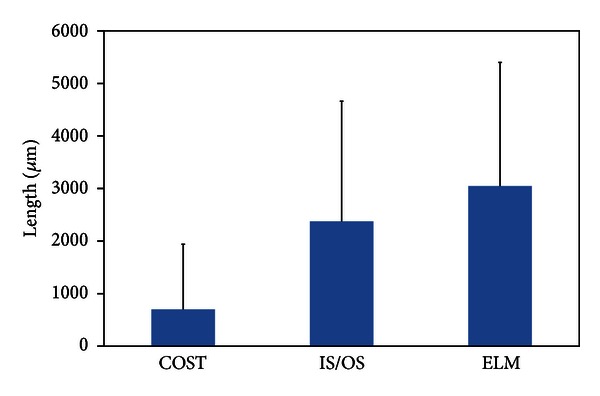
Mean lengths of the cone outer segment tips (COST), photoreceptor inner and outer segment (IS/OS) junction, and external limiting membrane (ELM) lines in 133 eyes with retinitis pigmentosa. The ELM length was significantly longer than the IS/OS or COST length, and the IS/OS length was significantly longer than the COST length (all *P* < 0.0001). Error bars represent one standard deviation from the mean.

**Figure 3:**
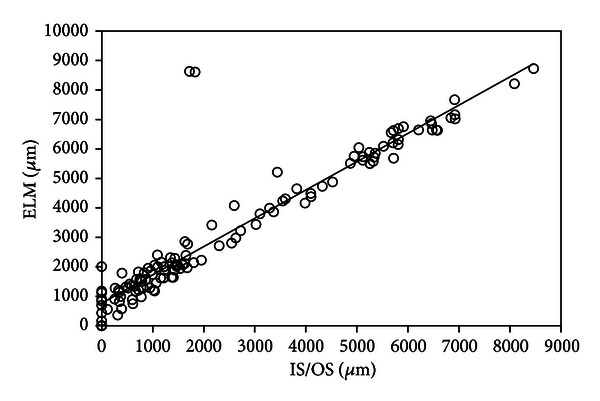
Correlation between the lengths of the photoreceptor inner/outer segment (IS/OS) junction and the external limiting membrane (ELM) lines in 133 eyes with retinitis pigmentosa. There is a significant positive correlation between the lengths of the IS/OS and ELM lines (*r* = 0.933, *P* < 0.0001). The solid line represents the linear regression curve (*y* = 768.13 + 0.96*x*).

**Figure 4:**
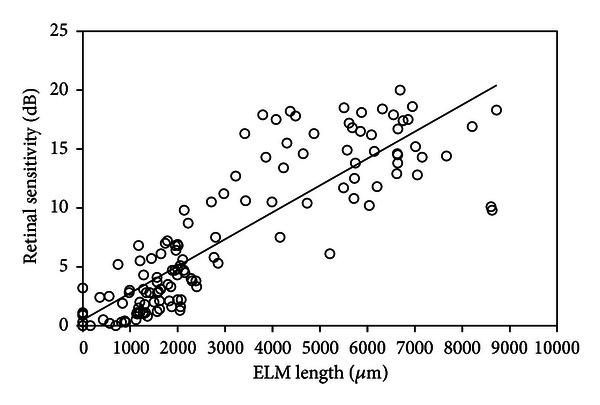
Correlation between the length of the external limiting membrane (ELM) line and the mean retinal sensitivity in 133 eyes with retinitis pigmentosa. There is a significant positive correlation between the length of the ELM line and the retinal sensitivity (*r* = 0.868, *P* < 0.0001). The solid line represents the linear regression curve (*y* = 0.488 + 0.002*x*).

Using SD-OCT, a recent study reported the results of examination of 133 patients with RP [9]. In this study, the lengths of the residual ELM, IS/OS, and COST lines were measured on SD-OCT images (Figure 1). The Micro Perimeter-1 (MP-1) (Nidek, Gamagori, Japan) was used to determine the mean retinal sensitivity at 24 locations covering the central 10° (Figure 1). The ELM length was significantly longer than the IS/OS or COST length, and the IS/OS length was significantly longer than the COST length (all *P* < 0.0001, Figure 2). In all subject eyes, the length was longest in the ELM, followed by the IS/OS and COST. The correlations among the ELM, IS/OS, and COST lengths were extremely significant (all *P* < 0.0001, Figure 3). The ELM, IS/OS, and COST lengths were significantly correlated with the mean retinal sensitivity and the visual acuity (all *P* < 0.01, Figure 4).

This study showed that the ELM, IS/OS, and COST lengths were highly correlated with each other and were significantly correlated with visual function. In each eye of all subjects, the length was the longest in the ELM, followed by the IS/OS and COST lines. These findings suggested that the shortening of the ELM, IS/OS, and COST lines is associated with each other and that retinal layer may become disorganized first at the COST line, followed by the IS/OS line and finally the ELM line. Wakabayashi et al. [10] also reported that the ELM length was significantly longer than the IS/OS length in RP patients. To exactly determine the time course of the changes in the ELM, IS/OS, and COST lines in RP patients, however, further follow-up longitudinal studies will be required.

Previous pathological studies of RP indicated that the earliest histopathological change in the rods is shortening of their outer segments, which can be visualized by immunocytochemistry using antirhodopsin [11]. The connecting cilium is a narrow stalk that joins the inner and outer segments of rods and cones. Several reports indicated that connecting cilia may be abnormal in RP patients [12, 13]. A structural or functional defect in the connecting cilium could result in decreased transport of newly synthesized proteins from the inner to the outer segment, resulting in the outer segment shortening and dysfunction that are characteristic of RP. Rod cell death in RP retinas is usually accompanied by changes in the neighboring cones, including outer segment shortening, cytoplasmic densification, axonal elongation, and, ultimately, cone cell death [14]. When all rods and most of the cones have died, the macula usually retains a monolayer of cone somata with very short or absent outer segments [14]. These pathological changes in RP are consistent with the OCT findings that retinal layer becomes disorganized first at the COST line, followed by the IS/OS line and finally the ELM line.

As for acute zonal occult outer retinopathy (AZOOR), Tsunoda et al. [15] reported that the COST line may be an early indicator of photoreceptor dysfunction in AZOOR. In their AZOOR cases, the COST line was always absent in the region of IS/OS abnormalities. Therefore, the authors concluded that the abnormality of the COST line may precede that of the IS/OS junction. In the region with the COST line abnormality in their AZOOR cases, the IS/OS junction was clearly observed, but the amplitude in focal macular electroretinograms and the retinal sensitivity were markedly reduced. Thus, the authors suggested that the photoreceptor dysfunction could be initially reflected by an absence or indistinctness of the COST line. However, care should be taken in the evaluation of the COST line because visibility of the COST line is dependent on the intensity and direction of the laser light that reaches the photoreceptor layer [16]. As an indicator of early cone photoreceptor dysfunction, the authors also found the foveal bulge which indicates a domelike appearance of the IS/OS junction due to an elongated cone outer segment at the fovea [15]. The foveal bulge could not be detected in their AZOOR cases with COST line abnormality and continuous IS/OS.

With respect to other retinal degenerative diseases, Lazow et al. [17] examined the structural changes across the transition zone between relatively normal retina and severely affected retina in choroideremia (CHM) and the Stargardt disease (STGD), and compared these to the transition zone in RP using SD-OCT. The ELM disappeared in a more affected location than did the IS/OS junction in all CHM and STGD patients, and these findings were similar to those in RP. These findings suggested that retinal layer may become disorganized at the IS/OS line, followed by the ELM line not only in RP but also in CHM and STGD. However, there were qualitative differences in the appearance of the transition zones. The distance between the disappearance point of the IS/OS line and the ELM line was significantly shorter in CHM and STGD, as compared with that in RP. In other words, the disappearances of the IS/OS and ELM lines were gradual (shallower slope) in RP, yet were nearly vertical (steeper slope) in CHM and STGD. Thus, the disappearances of the IS/OS junction and ELM were much more abrupt in CHM and STGD than in RP.

As for retinal impairment due to retinal detachment (RD), Wakabayashi et al. [18] evaluated foveal microstructural changes in eyes with anatomically successful repair of rhegmatogenous RDs using SD-OCT. In their study, no eyes had a disrupted ELM with an intact IS/OS junction, suggesting that mechanical splitting of the photoreceptor outer segments and RPE adhesion in RDs initially damages the photoreceptor outer segment layer and then causes degenerative changes that reach the photoreceptor cell bodies. As for evidence supporting this suggestion, several experimental studies have reported that the degenerated photoreceptor outer segment and the decreased outer segment length occur immediately after RDs [19, 20]. Apoptotic changes in the photoreceptor cells occur soon after detachment [21], followed by progressive loss of photoreceptors in eyes with long-standing RDs [22].

With respect to epiretinal membrane (ERM), Shimozono et al. [23] studied 50 eyes with idiopathic ERM using SD-OCT. There were no eyes with ELM disruption. The IS/OS junction also retained its continuity in all cases, while the COST line was disrupted in 48%. The authors speculated that the tractional force generated by ERM can alter the interface between the outer segment tips and the RPE without severely damaging the outer segment itself or photoreceptor cell bodies. In contrast, in more vision-threatening diseases such as age-related macular degeneration, the COST line is almost totally deteriorated, and the IS/OS junction, and even the ELM are disrupted in many cases [24]. Thus, there should be a hierarchy of vulnerability among the 3 lines; the COST line, the IS/OS junction and the ELM can be disrupted when mild, moderate, and severe photoreceptor damage, respectively, is caused [23].

## 3. Photoreceptor Restoration on Optical Coherence Tomographic Image

For the image assessment of the photoreceptor restoration on the OCT images, a lot of studies using OCT have reported on the relationship between the restoration of IS/OS junction and the recovery of the visual acuity after successful macular hole (MH) closure [25–30]. Using TD-OCT, it has been reported that the presence of the IS/OS line on the OCT images is correlated with the recovery of good vision after MH surgery and is essential for normal visual function [28].

After development of SD-OCT, SD-OCT images were analyzed in terms of the IS/OS junction and the ELM after idiopathic MH surgery. Ooka et al. [25] studied 43 eyes before and 1, 3, and 6 months after MH surgery. After MH surgery, the IS/OS or ELM line is restored from perifoveal region toward center of the closed MH on the OCT images. Therefore, measuring the length of defect in the IS/OS or ELM line can be useful in estimating the retinal restoration after MH surgery. The results of this study indicated that the length of the IS/OS junction defect was significantly correlated with the length of the ELM defect at all preoperative and postoperative times and that the restoration of the ELM was earlier than that of the IS/OS junction at all times and in all eyes. None of the eyes had a complete restoration of the IS/OS junction without a complete recovery of the ELM. These findings suggest that the restoration of the ELM is closely associated with that of the IS/OS junction, and the integrity of the ELM is necessary for the restoration of the IS/OS junction. At all postoperative times, the lengths of both the IS/OS and ELM defects were significantly correlated with both the visual acuity and the foveal sensitivity measured using MP-1. The restoration of the IS/OS junction and the ELM may reflect the morphological and functional recovery of the foveal photoreceptors in surgically closed MHs.

**Figure 5:**
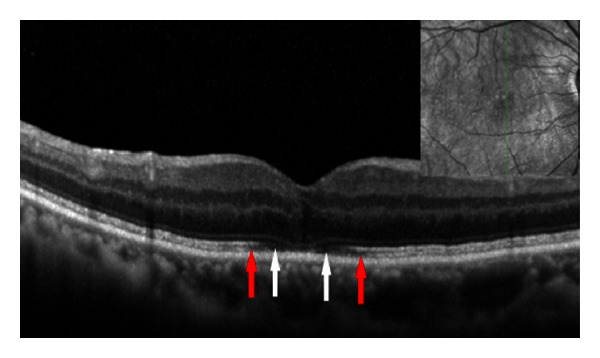
Spectral-domain optical coherence tomographic (SD-OCT) image of an eye 3 weeks after macular hole repair (72-year-old woman). SD-OCT image of a vertical scan was obtained. In addition to photoreceptor inner/outer segment (IS/OS) junction line, the external limiting membrane (ELM) and cone outer segment tips (COST) lines are clearly seen on the SD-OCT image. Red arrows indicate end points of the COST line. White arrows indicate end points of the IS/OS line. The ELM line has been almost restored. Note that the defect length was longest in the COST, followed by the IS/OS and ELM. The direction of photoreceptor restoration is from inside toward outside.

With respect to the COST line, it was reported that distinct COST line was first seen at 6 months after MH surgery [30]. On the other hand, distinct ELM and IS/OS lines were reported to be first seen at 1 month after surgery [30]. Itoh et al. [26] studied 51 eyes with MH before and 1, 3, 6, 9, and 12 months after MH surgery. The postoperative mean length of COST line defect progressively decreased, and the appearance of the COST line recovery began at the perifoveal region and progressed toward the center of the closed MH. The length of the COST line defect was significantly correlated with the visual acuity at 1, 3, 6, 9, and 12 months postoperatively. The recovered COST line was observed only in eyes with the intact IS/OS junction and ELM line. The length of COST line defect was significantly longer than that of the IS/OS or ELM line defect (both *P* < 0.01). Taken together with the results of the previous study [25], retinal layer becomes restored first at the ELM, followed by the IS/OS and finally the COST after MH surgery (Figure 5).

With respect to retinal restoration after ERM surgery, Shimozono et al. [23] studied 50 eyes that underwent vitrectomy for idiopathic ERM. There were no eyes with ELM disruption preoperatively and postoperatively. At baseline, the IS/OS junction retained its continuity in all cases, while the COST line was disrupted in 48%. The disruption of the IS/OS junction and the COST lines temporarily increased at 1 month postoperatively and decreased to near the baseline level thereafter. Postoperatively, defect lengths of IS/OS and COST lines were significantly correlated with the visual acuity.

As for retinal restoration after RD surgery, Wakabayashi et al. [18] evaluated foveal microstructural changes in eyes with anatomically successful repair of rhegmatogenous RDs using SD-OCT. In preoperative macula-off eyes, the postoperative visual acuity was significantly correlated with the integrity of the photoreceptor IS/OS and ELM lines detected by SD-OCT postoperatively. During the postoperative follow-up period, the IS/OS junction became restored in 64% of the eyes with the disrupted IS/OS junction and the continuous ELM line at the postoperative initial examination. In any eyes with the disrupted IS/OS and ELM at the initial examination, the photoreceptor layer did not become completely restored during the follow-up period. Thus, the authors concluded that the postoperative preservation of the ELM may predict the subsequent restoration of the photoreceptor layer in RD patients [18].

Regarding the histopathological findings of the detached retina after retinal reattachment, previous experimental studies have proposed that atrophy of the photoreceptors occurring soon after retinal reattachment may be irreversible in eyes with extended period of RD [19, 20, 22, 31]. However, the atrophy can stop or reverse in eyes with short time period of RD [19, 20, 22, 31]. Guérin et al. [19] investigated the recovery process of photoreceptor outer segments after retinal reattachment using the animal model of RD. RD leads to a reduction in photoreceptor outer segment absolute length. Increasing time of retinal reattachment is positively correlated with an increase in outer segment absolute length. Rod and cone outer segments regained approximately 40% of their control lengths after a 2-week reattachment period. By 30 days of reattachment, rod outer segments had regained 72% of their normal length, and cone outer segments had regained approximately 48%. After 150 days of reattachment, photoreceptor outer segment mean length was not statistically different from control areas. This histopathological finding of increase in the photoreceptor outer segment length after retinal reattachment suggested the direction of photoreceptor restoration from inside toward outside, being consistent with the SD-OCT findings after the repair of RD [18].

The process of the photoreceptor restoration after MH or RD surgery seems to be exact opposite of the process of photoreceptor impairment in RP or other retinal degenerative diseases. It is known that the photoreceptors continuously add and shed discs of the outer segments [19]. This renewal of the outer segment has been suggested to be related to the recovery of the length of the foveal photoreceptor outer segments [19]. The ELM is the first structure to recover after MH closure [25, 27, 29]. The ELM is considered to consist of zonular adherence between photoreceptor inner segment and the Müller cell processes, and there is no zonula occludens in ELM [32]. Thus, ELM is macromolecule-impermeant intermediate junction, and microscopic particles such as horseradish peroxidase can pass through the intercellular space of ELM. ELM is thought to have a supportive function in maintaining the alignment and orientation of the photoreceptor [33]. Therefore, restoration of the ELM may be necessary for sequential photoreceptor outer segment repair, although the reason why the structure of ELM is most preserved in various retinal disease remains unclear. A continuous ELM has been considered to be a sign of intact photoreceptor cell bodies and the Müller cells, and the IS/OS junction or COST rarely recovered without a recovery of the ELM [25–27, 29]. Reconstruction of the foveal ELM line in the early postoperative period can help to predict subsequent restoration of the foveal photoreceptor layer and the potential for better visual outcomes after MH surgery [29]. Bottoni et al. [27] reported that an intact outer nuclear layer at the fovea also seems to be necessary to achieve a complete restoration of the photoreceptor microstructure.

## 4. Histology and Optical Coherence Tomographic Image

Among three highly reflective lines depicted inside the RPE on the SD-OCT image, the ELM is considered to consist of zonular adherence between photoreceptor inner segment and the Müller cell processes [32]. The ELM line typically is thinner and much fainter than the other two lines. The second line has been commonly ascribed to the boundary between the inner segments and outer segments of the photoreceptors (IS/OS) [3]. The COST line, also known as the Verhoeff membrane [16], is considered to be attributed to scattering from the tips of the cone outer segment, which is shorter than the rod outer segment, in the region of interdigitation between the photoreceptor outer segment and the RPE cell processes that extend into the outer segment layer.

Most recently, Spaide and Curcio [3] evaluated the validity of commonly used anatomical designations for these hyperreflective lines. A scale model of outer retinal morphology was created using published information for direct comparison with SD-OCT scans. Their analysis showed a high likelihood that the SD-OCT lines attributed to the ELM (the first, innermost line) are correctly attributed. Comparative analysis showed that the second line, often attributed to the boundary between inner and outer segments of the photoreceptors, actually aligns with the ellipsoid portion of the inner segments. The third line corresponded to an ensheathment of the cone outer segments by apical processes of the RPE in a structure known as the contact cylinder.

Further studies regarding comparison between OCT images *in vivo* with histological correlative will be needed to understand what the change of OCT finding actually means. Only by making an accurate assessment of OCT findings, we can understand the precise changes of the photoreceptor *in vivo* in various retinal diseases.

## 5. Conclusion

Three highly reflective lines in the outer retina, which are the ELM, IS/OS and COST, can serve as hallmarks for the evaluation of photoreceptor condition. In RP patients, the ELM, IS/OS, and COST lines are shortened with the progression of the disease. The shortening of the ELM, IS/OS, and COST lines is significantly associated with each other. In each eye, the line length was longest in the ELM, followed by the IS/OS, and COST, suggesting that retinal layer becomes disorganized first at the COST line, followed by the IS/OS line and finally the ELM line [9]. On the other hand, retinal layer becomes restored first at the ELM, followed by the IS/OS and finally the COST after MH surgery. Taken together, there may be a directionality of the photoreceptor impairment or restoration on OCT image. Further studies with the use of high-resolution images of OCT should lead to understanding a more precise process of the photoreceptor impairment or restoration in various retinal diseases.
